# Weather associations with physical activity, sedentary behaviour and sleep patterns of Australian adults: a longitudinal study with implications for climate change

**DOI:** 10.1186/s12966-023-01414-4

**Published:** 2023-03-14

**Authors:** Ty Ferguson, Rachel Curtis, Francois Fraysse, Timothy Olds, Dorothea Dumuid, Wendy Brown, Adrian Esterman, Carol Maher

**Affiliations:** 1 Alliance for Research in Exercise, Nutrition and Activity (ARENA), University of South Australia, Adelaide, SA Australia; 2 School of Human Movement and Nutrition Sciences, University of Queensland, Brisbane, QLD Australia

**Keywords:** Physical activity, Sedentary behaviour, Sleep, Weather, Movement behaviours, 24-h day, Time use, Temperature, Rainfall, Wind speed

## Abstract

**Background:**

Weather is a potentially important influence on how time is allocated to sleep, sedentary behaviour and physical activity across the 24-h day. Extremes of weather (very hot, cold, windy or wet) can create undesirable, unsafe outdoor environments for exercise or active transport, impact the comfort of sleeping environments, and increase time indoors. This 13-month prospective cohort study explored associations between weather and 24-h movement behaviour patterns.

**Methods:**

Three hundred sixty-eight adults (mean age 40.2 years, SD 5.9, 56.8% female) from Adelaide, Australia, wore Fitbit Charge 3 activity trackers 24 h a day for 13 months with minute-by-minute data on sleep, sedentary behaviour, light physical activity (LPA), and moderate-to-vigorous physical activity (MVPA) collected remotely. Daily weather data included temperature, rainfall, wind, cloud and sunshine. Multi-level mixed-effects linear regression analyses (one model per outcome) were used.

**Results:**

Ninety thousand eight hundred one days of data were analysed. Sleep was negatively associated with minimum temperature (-12 min/day change across minimum temperature range of 31.2 °C, *p* = 0.001). Sedentary behaviour was positively associated with minimum temperature (+ 12 min/day, range = 31.2 oC, *p* = 0.006) and wind speed (+ 10 min/day, range = 36.7 km/h, *p*< 0.001), and negatively associated with sunshine (-17 min/day, range = 13.9 h, *p* < 0.001). LPA was positively associated with minimum temperature (+ 11 min/day, range = 31.2 °C, *p* = 0.002), cloud cover (+ 4 min/day, range = 8 eighths, *p* = 0.008) and sunshine (+ 17 min/day, range = 13.9 h, *p* < 0.001), and negatively associated with wind speed (-8 min/day, range = 36.7 km/h, *p* < 0.001). MVPA was positively associated with sunshine (+ 3 min/day, range = 13.9 h, *p* < 0.001) and negatively associated with minimum temperature (-13 min/day, range = 31.2 oC, *p* < 0.001), rainfall (-3 min/day, range = 33.2 mm, *p* = 0.006) and wind speed (-4 min/day, range = 36.7 km/h, *p* < 0.001). For maximum temperature, a significant (*p* < 0.05) curvilinear association was observed with sleep (half-U) and physical activity (inverted-U), where the decrease in sleep duration appeared to slow around 23 °C, LPA peaked at 31 oC and MVPA at 27 °C.

**Conclusions:**

Generally, adults tended to be less active and more sedentary during extremes of weather and sleep less as temperatures rise. These findings have the potential to inform the timing and content of positive movement behaviour messaging and interventions.

**Trial registration:**

The study was prospectively registered on the Australian New Zealand Clinical Trial Registry (Trial ID: ACTRN12619001430123).

**Supplementary Information:**

The online version contains supplementary material available at 10.1186/s12966-023-01414-4.

## Introduction

At any time of the day, a person is engaged in one of three movement behaviours; sleeping, being sedentary, or being active [1]. The relative distribution of these behaviours has impacts on health. Insufficient physical activity or sleep, and excess sleep or sedentary behaviour are associated with poor health outcomes, including increased risk of chronic illness and reduced life expectancy [2–5]. When considering day-to-day changes in movement behaviours, each is intrinsically linked, whereby changes in one behaviour result in equal and opposite changes across the remaining behaviours [6]. The time allocated to each behaviour in a 24-h day is influenced by both internal factors such as motivation, fatigue, and physical health, and external factors, including work or family commitments, physical environment, resource availability and the weather [7–9].

The weather we experience each day is the result of a complex and ever-changing system of atmospheric variables [10]. Weather conditions can either afford or limit opportunities to participate in movement behaviours. This may be directly, for example through impacting thermoregulation, [11] or indirectly by changing the usability and safety of the physical environment [12]. Whilst weather is not an acutely modifiable factor, understanding its association with movement behaviours is valuable for identifying potential conditions where less favourable movement behaviour patterns occur.

Extremes of weather (i.e. too hot, cold, windy or wet) reduces mobility and engagement with our physical environment [12, 13]. This may limit opportunities for physical activity, including outdoor exercise, recreation, and safe active transport, reduce the comfort of sleeping environments, and more time may be spent indoors where sedentary pursuits are common (i.e. sitting, watching TV, using computers and mobile devices) [14]. Current literature suggests that in general adults sleep less as temperatures rise, are more sedentary when rainfall is higher or temperatures are lower, and are less active during extremes of temperature, and during higher rainfall and wind speeds [15–19]. There is currently no clear association between movement behaviours and cloud cover or daily sunlight volume. The direction of the associations are more generalisable than specific magnitudes, as weather is regionally specific [15]. Weather patterns are influenced by a region’s distance from the equator, daylight hours, height above sea level, topography and proximity to the ocean [20].

There are several limitations to the current body of evidence for weather-based research. Broadly, a large portion of studies has focused on seasonal changes, which provides only a low-resolution comparison of weather conditions in specific timepoints across the year [15]. Additionally, studies have mostly used cross-sectional designs with subjectively measured movement behaviours, and often explore movement behaviours in isolation, e.g. physical activity only [15]. To our knowledge, no studies anywhere in the world have used a longitudinal design over a full 12-month seasonal cycle, to capture 24-h movement behaviour. The current study aims to fill these gaps by exploring the associations between weather conditions (maximum temperature, minimum temperature, rainfall, wind speed, cloud cover and sunshine) and daily device-measured movement behaviours (sleep, sedentary behaviour, light physical activity and moderate-to-vigorous physical activity) and to comment on how these associations fit with climate change projections. The findings may assist with the timing and design of movement behaviour interventions.

## Methods

### Study design

Data collected from the *Annual Rhythms In Adults' lifestyle and health* (ARIA) prospective cohort study were used in this analysis. ARIA collected data on body weight, 24-h movement behaviours, dietary patterns and wellbeing in a cohort of Australian adults over a 13-month period. Further details of the study protocol have been published elsewhere [21].

Ethics approval was provided by the University of South Australia Human Research Ethics committee (Protocol number: 201901). The study was prospectively registered on the Australian New Zealand Clinical Trial Registry (Trial ID: ACTRN12619001430123).

### Setting and participants

Three hundred seventy-five community-based adults were recruited from the greater metropolitan area of Adelaide, South Australia for the ARIA study. They were recruited in two ways: (1) parents of children enrolled in a separate cohort study called *Life on Holidays* [22] (cohorts 1 and 2), or (2) parents of primary school children recruited from the community through general advertising (i.e. social media posts, paid digital advertisements, community notice boards) (cohort 3).

Enrolment occurred in two waves: cohort 1 participants commenced on 1^st^ December 2019, cohorts 2 and 3 commenced on 1^st^ December 2020. Each wave remained in the study until the end of the following December (31^st^ December 2020 and 31^st^ December 2021 respectively), totalling 13 months in the study.

Inclusion criteria were: age 18 to 65 years; a parent/guardian of a child enrolled in Life on Holidays study or a parent/guardian of child aged 5 to 12 years; residing in the greater metropolitan Adelaide area; having access to a Bluetooth-enabled mobile phone or computer and home internet; being proficient in English; and being ambulant. Exclusion criteria were: being pregnant; having an implanted electronic medical device; or receiving treatment for, or experiencing, any life-threatening condition which impacted daily lifestyle and health.

A researcher visited each eligible and consenting participant at their home between August and November in the year of commencing the study. At this visit baseline height was measured and participants completed a baseline survey containing demographic, health and lifestyle items. Participants were provided with a Fitbit Charge 3 fitness tracker and Fitbit Aria body weight scale (Aria 2 or Aria Air scale, Fitbit Inc., San Francisco, CA, USA).

During the study period, participants used the Fitbit devices daily and completed eight follow-up surveys of wellbeing and diet (not used in the current analysis). Participants did not require any face-to-face session after the baseline visit, unless technical support was required. On completion of the study, participants received an honorarium of $100 and were able to keep the Fitbit Charge 3 and Aria scale.

### Variables

#### Daily movement behaviour patterns

Device-measured minute-by-minute movement behaviour data were collected using a wrist-worn Fitbit Charge 3 activity tracker (Fitbit Inc., San Francisco, CA, USA). The device was worn on the participants’ non-dominant wrist, 24 h a day (except whilst charging or during water activities). Participants were asked to sync data to the linked Fitbit user account frequently (at least every 5 days), so that data could be collected remotely via purpose-built software called “Fitnesslink” (Portal Australia, Adelaide, Australia).

Each minute was classified as either sleep, sedentary behaviour, light physical activity, moderate or vigorous physical activity, or non-wear time, using Fitbit's proprietary algorithm. Fitbit devices have shown acceptable validity for sleep duration (compared to polysomnography: Charge 2 sensitivity = 0.96, specificity = 0.61; [23] Flex *r* = 0.97 [24]), sedentary behaviour (compared to ActivPAL: Charge 3 ICC = 0.94 (95% CI: 0.92–0.96 [25])) and moderate-to-vigorous physical activity (compared to Actigraph: Charge 2 ICC = 0.69 (95% CI: 0.35–0.87) [26], Flex *r* = 0.73 [27]).

#### Weather

Adelaide is the capital city of South Australia with a population of approximately 1.4 million people. The metropolitan area is situated on a western coastline with flat plains extending toward steep hills in the eastern and southern regions (34° latitude and 138° longitude) [28]. Adelaide's climate is considered temperate (Köppen classification), [29] and is characterised by four distinct seasons, including cold, wet winters and warm, dry, low humidity summers [30]. Average weather data during the study are summarised in Table 1. Daily weather data for Adelaide were collected from the Australian Data Archive for Meteorology [31]. Weather variables were reported as the average of data from five major weather stations spread geographically across the Adelaide metropolitan area. Maximum temperature (^o^C), minimum temperature (^o^C), rainfall (mm) and sunshine (defined as “bright” sunshine, which is less than the amount of visible sunlight per day, e.g. sunrise and sunset would be below the threshold for “bright” sunshine) [32] were reported as a single daily value. Wind speed (km/h) was reported as maximum gust, and at 9am and 3 pm daily, with an average of the three values used to derive an overall daily value. Relative humidity (%) and cloud cover (eighths) were reported twice daily (9am and 3 pm) with an average of the two used to derive an overall daily value. Average temperature (^o^C) was calculated as the mean of maximum and minimum temperatures. Apparent temperature (^o^C) was calculated using the Steadman formula [33] incorporating average temperature, humidity, wind speed and atmospheric pressure (hPa).

**Table 1:**
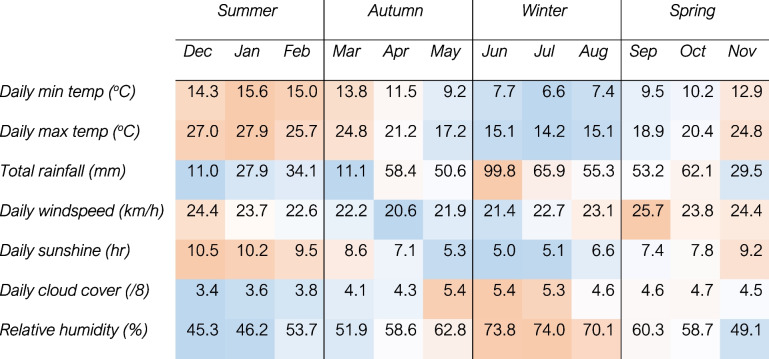
Average monthly weather during study period

Note: Colour gradient represents lowest month (darkest blue) to highest month (darkest orange) for each variable. Each column is the average of the years over which the study was conducted, i.e. January to November columns are the average of 2020 and 2021, and December is the average of 2019, 2020 and 2021

*Max* maximum, *min* minimum, *temp* temperature

#### Demographic characteristics

At baseline, participants reported their sex, date of birth, marital status, number of children in household, smoking status, presence of chronic conditions, highest education level, combined gross household income and hours of work per week outside the home. They also self-reported their sleep chronotype using a single-item from the Morningness-Eveningness questionnaire [34].

### Statistical analysis

Daily total minutes of sleep, sedentary behaviour, light physical activity and moderate-to-vigorous physical activity were calculated for each participant day. A valid day was defined as 18 h or more of wear-time including a period of sleep. Moderate-to-vigorous physical activity was calculated as the sum of moderate physical activity and vigorous physical activity daily totals. Days on which a participant reported being away from home on vacation were excluded from analyses. Participants were included if they had at least 2 valid days of data to ensure repeat measurement.

Prior to performing the primary statistical analyses, independent weather variables were compared for collinearity (Table 2). Where a high correlation (≥ 0.80) was present, only one of the paired variables was included in the primary analyses. Specifically, maximum temperature was retained as it was the only common variable across all high correlation pairings. Six weather variables were retained from the original nine, these were minimum temperature, maximum temperature, rainfall, wind speed, sunshine and cloud cover.

**Table 2:** Pearson correlation coefficients between weather variables

	*Min temp*	*Max temp*	*Avg temp*	*App temp*	*Rainfall*	*Sunshine*	*Humidity*	*Wind speed*	*Cloud cover*
*Min temp*									
*Max temp*	0.77								
*Avg temp*	**0.92**	**0.96**							
*App temp*	**0.90**	**0.93**	**0.97**						
*Rainfall*	-0.11	-0.31	-0.24	-0.24					
*Sunshine*	0.11	0.48	0.35	0.32	-0.22				
*Humidity*	-0.49	**-0.82**	-0.73	-0.61	0.39	-0.50			
*Wind speed*	0.22	-0.03	0.07	-0.11	0.22	-0.10	-0.04		
*Cloud cover*	-0.03	-0.38	-0.25	-0.23	0.20	-0.78	0.44	0.18	

Bold indicates a high correlation (≥ ± 0.80)

*Avg* Average, *app* Apparent, *max *Maximum*, min* Minimum, *temp* Temperature

Independent vs dependent variable scatterplots of raw data were generated in R version 4.1.3 (R Foundation for Statistical Computing, Vienna, Austria) and presented as smoothing lines with 95% confidence intervals using generalised linear models for linear relationships and generalised additive models for quadratic relationships. A quadratic relationship was observed between maximum temperature and movement behaviour variables; to account for this relationship a variable of maximum temperature squared was included in the analyses.

Multi-level mixed-effects linear regression analyses were performed using Stata 17 (StataCorp, College Station, TX, USA) with statistical significance set at 0.05. Multilevel modelling with random intercepts was used to adjust for the non-independence of the data and to account for nesting of repeated measures within individuals, individuals within families, and families within waves. The movement behaviours were the dependent variables (one per model). The weather variables were included as fixed effects (all included in the same model). The regression coefficients for the weather variables were used to identify the difference in daily minutes of movement behaviours associated with an increase of one degree of temperature, one millimetre of rainfall, one kilometre per hour of wind speed, an eighth of cloud cover, and one minute of sunshine.

## Results

### Participants

Movement behaviour data were available for 368 of the initial 375 enrolled (see Table 3). Participants were predominantly mid-aged (mean = 40 years [SD = 6 years], range = 27 to 65 years), overweight or obese (34% and 35% respectively), married or in de facto relationships (85%), and had two or more children in their household (90%). Almost half the participants were educated to university level (48%), worked full time equivalent hours (46%), worked in managerial or professional occupations (40%) and had a household income between $100,000 and $200,000 (48%).

**Table 3:** Participant demographics (*n* = 368)

	**Mean**	**(SD)**
**Age (years)**	40.2	(5.9)
**Weight (kg)**	84.0	(20.5)
**Height (cm)**	170.4	(9.5)
	**N**	**(%)**
**Sex**		
Female	209	(56.8)
Male	159	(43.2)
**Weight status**		
Underweight	1	(0.3)
Normal	114	(31.0)
Overweight	124	(33.7)
Obese	129	(35.1)
**Smoker**	34	(9.2)
**Aboriginal and Torres Strait Islander peoples**	4	(1.1)
**Born in Australia**	279	(75.8)
**Marital status**		
Never married	26	(7.1)
Married/de facto	313	(85.1)
Separated, divorced or widowed	29	(7.8)
**Chronic illness**		
None	163	(44.3)
Single	107	(29.1)
Multiple	98	(26.6)
**Self-reported sleep chronotype**		
Definitely a morning type	82	(22.3)
More a morning type than evening type	122	(33.2)
More an evening type than morning type	111	(30.2)
Definitely an evening type	53	(14.4)
**Adults in household**		
One	38	(10.3)
Two	305	(82.9)
Three	17	(4.6)
Four or more	7	(1.9)
**Children in household**		
One	36	(9.8)
Two	195	(53.0)
Three	95	(25.8)
Four or more	42	(11.5)
**Education**		
Year 10 or less	17	(4.6)
Year 11—12	49	(13.3)
Certificate/Diploma	125	(34.0)
University degree	177	(48.1)
**Occupation**		
Managerial and professional	148	(40.2)
Technical and clerical	77	(20.9)
Community, personal service, sales	67	(18.2)
Machinery operators, drivers, labourers	14	(3.8)
No job/other	62	(16.8)
**Household income (Australian dollars)**		
< $50,000	37	(10.1)
Between $50,000 and $99,999	111	(30.2)
Between $100,000 and $199,999	176	(47.8)
≥ $200,000	44	(12.0)
None	55	(14.9)
< 15	25	(6.8)
15–35	119	(32.3)
36 +	169	(45.9)

### Movement behaviour data

Data from 90,801 valid days were analysed, with an average of 247 (SD = 113) valid days per participant (see Supplementary Fig. 1 for distribution of valid days per participant). On a day-by-day basis, the average percentage of participants with valid data was 64% (SD = 6.6%) with the daily percentage declining gradually over the 13-month study period (first month = 73%, thirteenth month = 51%). Across all valid days, participants' daily averages for movement behaviours were 8 h and 10 min of sleep (SD = 89 min), 10 h and 19 min of sedentary behaviour (SD = 120 min), 4 h and 59 min of light physical activity (SD = 93 min) and 32 min of moderate-to-vigorous physical activity (SD = 38 min).

### Associations between weather and movement behaviours

**Fig. 1:**
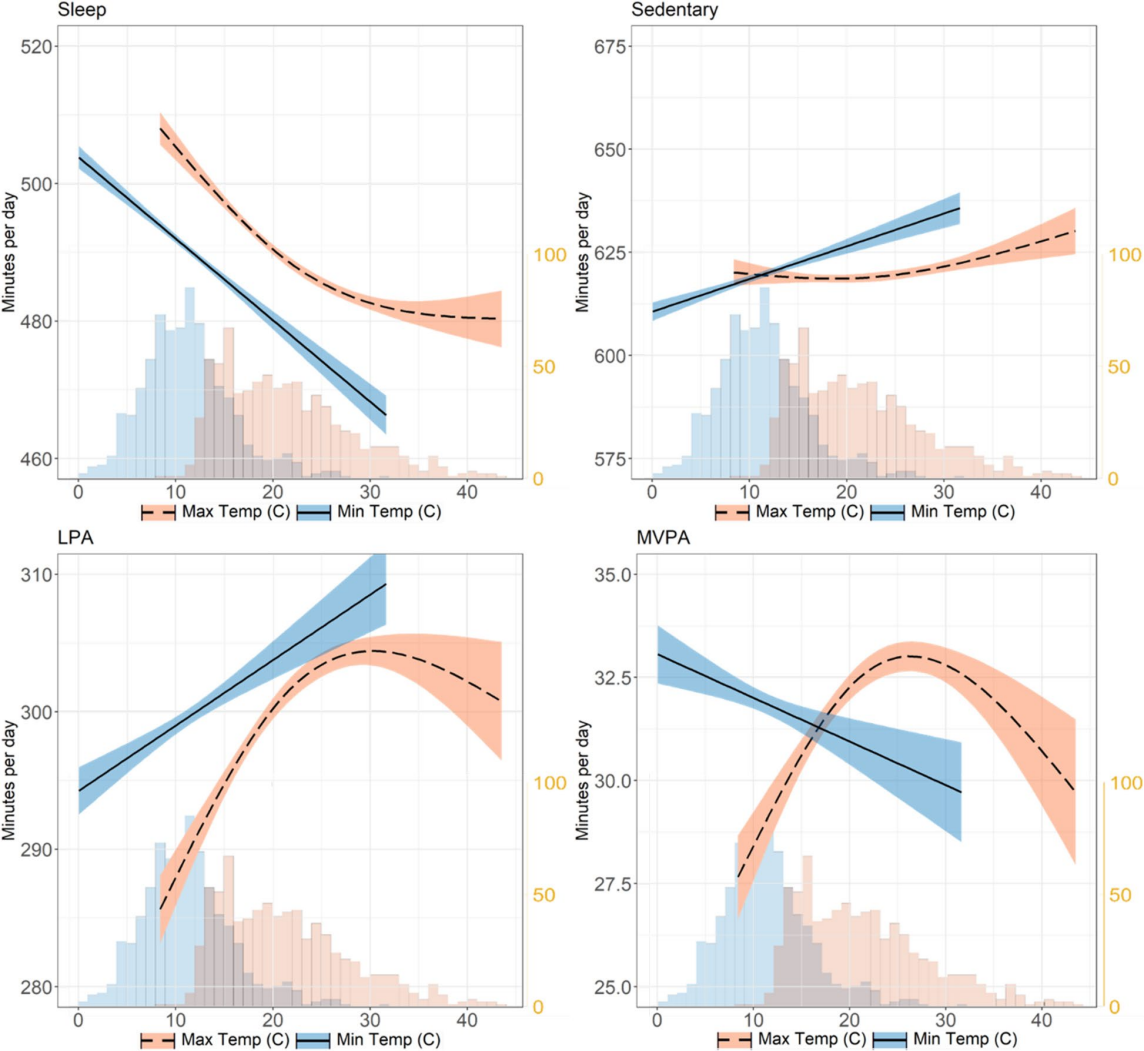
Fitted line plot of daily maximum and minimum temperatures vs movement behaviours. Notes: Max Temp = maximum daily temperature (^o^C), Min Temp = minimum daily temperature (.^o^C). Coloured area represents 95% confidence intervals. Generalised additive models used to produce Max Temp smoothed line and generalised linear models used to produce Min Temp smoothed line. Right y-axis represents histogram showing frequency of temperature at 1 °C intervals for maximum daily temperature (blue) and minimum daily temperature (orange)

The relationships between temperature (maximum and minimum) and observed raw movement behaviour data are shown in Fig. 1. For maximum temperature, the non-linear relationship is represented using generalised additive models for line fitting. Notably, both light physical activity and moderate-to-vigorous physical activity increase with temperature from 9 oC towards a peak at approximately 31 oC and 27 oC respectively, before decreasing as temperature increases to the upper limit of 44 oC. The optimal 10 oC range for maximum temperature appears to be 25–35 oC for light physical activity and 22–32 oC for moderate-to-vigorous physical activity, giving an overlap of 25–32 oC for both activity types to be optimised.

Results of multi-level mixed-effects linear regression analyses are presented in Table 4. Sleep, light physical activity and moderate-to-vigorous physical activity were all associated with both maximum temperature and maximum temperature squared, suggesting a curvilinear relationship. Sleep was also negatively associated with minimum temperature (-12 min/day change across minimum temperature range of 31.2 °C). Sedentary behaviour was positively associated with minimum temperature and wind speed (+ 12 min/day, range = 31.2 °C and + 10 min/day, range = 36.7 km/h respectively), and negatively associated with sunshine (-17 min/day, range = 13.9 h). Light physical activity was positively associated with minimum temperature (+ 11 min/day, range = 31.2 oC), cloud cover (+ 4 min/day, range = 8 eighths) and sunshine (+ 17 min/day, range = 13.9 h), and negatively associated with wind speed (-8 min/day, range = 36.7 km/h). Moderate-to-vigorous physical activity was positively associated with sunshine (+ 3 min/day, range = 13.9 h), and negatively associated with minimum temperature (-13 min/day, range = 31.2 oC), rainfall (-3 min/day, range = 33.2 mm), and wind speed (-4 min/day, range = 36.7 km/h).

**Table 4:**
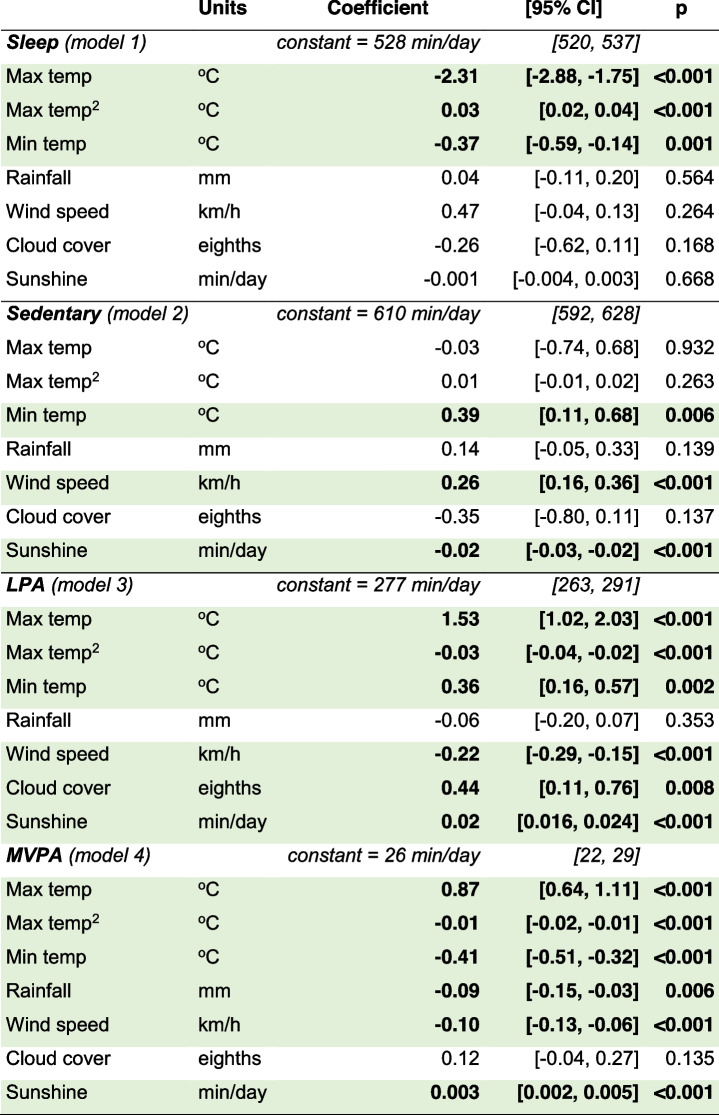
Associations between movement behaviours and weather

Results of multi-level mixed-effects linear regression analyses (one model per movement behaviour). Models adjusted for nesting of observations within participants, within families, within study waves. Bold and green shading indicates *p* < 0.05

*LPA*  Light physical activity, *max temp* Maximum temperature, *max temp*^2^ Maximum temperature squared, *min temp* Minimum temperature, *MVPA* Moderate-to-vigorous physical activity

## Discussion

This study explored the associations between daily weather conditions and device-measured movement behaviours using longitudinal data across a 13-month period. Several significant associations were observed. Sleep was inversely associated with minimum temperature, sedentary behaviour was inversely associated with sunshine, and positively associated with minimum temperature and windspeed. Light physical activity was inversely associated with windspeed, and positively associated with minimum temperature, cloud cover and sunshine. Moderate-to-vigorous physical activity was inversely associated with minimum temperature, rainfall, and windspeed, and positively associated with sunshine. For maximum temperature associations, an inverted-U curvilinear relationship was observed with both light and moderate-to-vigorous physical activity, and an exponential decay relationship with sleep (i.e. decreasing slope as maximum temperature increases). The directions of the associations observed in this study were largely consistent with previous literature.

### Sleep

Sleep duration decreased as minimum temperature increased with an estimated reduction of 12 min across the 30 oC temperature range experienced by participants. This magnitude of change is consistent with a large global study in which a 9 min reduction was observed over the same temperature range [16]. Additionally, comparable reductions in sleep were observed in studies using self-reported sleep measures [35]. Participants in this study were, on average, within recommended sleep durations of 7 to 9 h, [36] and the modelled range of sleep duration change would not have moved the average person outside these recommendations. However, it is important to consider that nightly sleep needs are person- and context-specific. Finally, the acute effect of accumulated sleep reduction over days or weeks of very high overnight temperatures are wide ranging, including reduced cognitive ability, increased stress responsivity, emotional distress and reduced well-being [37]. The ability to maintain favourable indoor temperatures is highly resource-dependent and the effect of high overnight temperatures is likely to be greater for poor and low-resourced populations [38].

### Physical activity

Light physical activity increased with minimum temperature, cloud cover and sunshine, and decreased as wind speed increased. These results are similar to the limited strength evidence in Turrisi et al.’s [15] review. No evidence was found for associations with cloud cover and sunshine in that review. Moderate-to-vigorous physical activity decreased as minimum temperature, rain and wind increased, and increased as sunshine increased. Combining this with the curvilinear relationship of maximum temperature, it appears ideal conditions were dry, calm, sunny days where maximum temperatures ranged from 22 °C-32 ^o^C. Turrisi et al [15]. reported similar results, with strong evidence for a curvilinear relationship with maximum temperature. Interestingly, the percentage of days where minimum recommended physical activity levels were achieved was relatively stable across the 20–40 oC maximum temperature range (51.9%-53.5% range across 5 oC groupings). However, there was a sharp decrease in the number of days when physical activity guidelines were met, when the maximum temperature was above 40 oC (44.9% vs 52.3% for days 35–40 oC). At the population level, this is a meaningful decline in moderate-to-vigorous physical activity on very hot days.

When considering the physical environment, extremes of weather have the potential to create unsafe environments for exercise, active transport and simply being outdoors [12]. Rain reduces the quality of surfaces and visibility, wind reduces stability and increases surface debris, radiant heat can raise temperatures of asphalt, concrete and metal surfaces to unsafe levels [12]. It is easier to protect oneself from the elements with clothing and equipment (i.e. umbrella), or by staying indoors when being lightly active (i.e. walking), however this becomes more challenging with more demanding forms of activity such as cycling or running without access to indoor exercise equipment. Risk of injury also increases with more vigorous activities in less controlled environments.

### Sedentary behaviour

The duration of sedentary behaviour increased as wind and minimum temperature increased, and decreased when sunshine increased. These findings are consistent with the moderate strength evidence reported by Turissi et al [15]. for temperature, and the limited strength evidence for windspeed [15]. No association between sedentary behaviour and rainfall or cloud cover is also consistent with Turissi et al.’s findings [15]. Interestingly, when looking at significant pairings of movement behaviours, seven involved sedentary behaviour and another movement behaviour, five of which were opposite association pairings. This suggests time allocation more commonly swaps between sedentary behaviour and the other movement behaviours. This swapping is supported by perceptions of extremes of weather as a barrier to physical activity, a negative correlate of sleep, and a positive correlate of sedentary behaviour [39–41].

### Strengths and limitations

To our knowledge, this is the first longitudinal study of one year duration that captures 24-h movement behaviours in an adult population. The dataset included device-measured movement behaviour and climatic data. This is a large, high-quality dataset for answering the research questions at hand. There are a few limitations to consider. The sample size limited the study’s power to explore potential participant sub-group differences. Data were collected in a single Australian city with a Mediterranean climate [29]. Thus, the findings are likely to be most applicable to other temperate climate regions (which includes parts of North and South America, Africa and Europe), and do not provide insight into extreme climates such as extremely cold or tropical climates. The population consisted of adults with primary-school-aged children, so adults’ weather-related behaviour may additionally reflect the needs of children in their care. Furthermore, data collection took place from 2019–2021, much of which was within the Coronavirus disease 2019 (COVID-19) pandemic period. Due to Australia’s strict quarantine approach, lifestyles of individuals residing in Adelaide were not harshly impacted by COVID-19, though there were periods in which working from home for non-essential workers was encouraged, and short periods of gym closures (less than one week durations, after an initial two-month shutdown).

### Implications for intervention design

Several implications for intervention and health promotion campaigns should be considered. Firstly, the weather may influence intervention effects. For example, if an intervention spans months or seasons with meaningfully different weather conditions, weather should be considered when discussing findings, or potentially be included in statistical analyses. At the person-specific level, an interesting opportunity exists to explore a strategy known as just-in-time adaptive interventions. These are study designs that adapt the amount of intervention to the individual’s context [42]. By leveraging longitudinal activity tracker data and combining this with weather forecasting, an opportunity exists to tailor health promotion messaging or intervention content to the individual at the specific time it is most needed. Future research in this area is warranted. Finally, community-level health promotion campaigns may be timed to target periods in the year, either by season or month, where less favourable weather conditions historically occur, i.e. promoting sleep and sleep hygiene in summer months, or ideas for “swapping” outdoor physical activity for indoor alternatives in winter and inclement weather.

### Implications in the context of climate change

At present, we are amid an unprecedented climate crisis [43]. Global temperatures are increasing, rainfall patterns are changing, and the frequency of extreme weather events is on the rise [43]. This is having far-reaching negative health consequences [38, 44–46]. As these patterns progress, the number of days each year where weather conditions are favourable for movement behaviours and outdoor environments are accessible for recreation is likely to decrease.

There are several options for mitigating the negative health effects of a changing climate, at both the individual level and as a society. Individually, a push toward increasing active transport has health and financial benefits to the individual, whilst also reducing a person’s CO_2_ emissions and contribution to traffic congestion [47]. Whilst this is a viable option, a caveat is that this type of activity is highly weather dependent, and viability is likely to decrease as climate change progresses. Another approach is extremes of weather action planning. Provision of freely accessible sheltered and indoor recreation areas is needed, especially in more extreme climate locations and for low-resourced populations [48].

## Conclusion

The results suggest modest significant associations between a range of weather variables and movement behaviours. In general, participants tended to be less active and more sedentary during extremes of weather and slept less as temperatures increased These findings have the potential to inform the timing and content of health promotion interventions at both the community and individual level, along with suggesting how health-related behaviours may be impacted over the coming decades by climate change.

## Supplementary Information

**Additional file 1.** 

**Additional file 2:** **Supplementary Table 1.** Descriptive daily weather statistics.

## Data Availability

Data used in the current study are available and may be obtained from the corresponding author upon reasonable request.

## Abbreviations

ARIA: Annual Rhythms In Adults’ lifestyle and health study
COVID-19: Coronavirus disease 2019
LPA: Light physical activity
MVPA: Moderate-to-vigorous physical activity
