# Supplementary figures and images for: Weather associations with physical activity, sedentary behaviour and sleep patterns of Australian adults: a longitudinal study with implications for climate change
Source: Int J Behav Nutr Phys Act. 2023 Mar 14;20:30. doi: 10.1186/s12966-023-01414-4 (PMC10012316; doi:10.1186/s12966-023-01414-4)

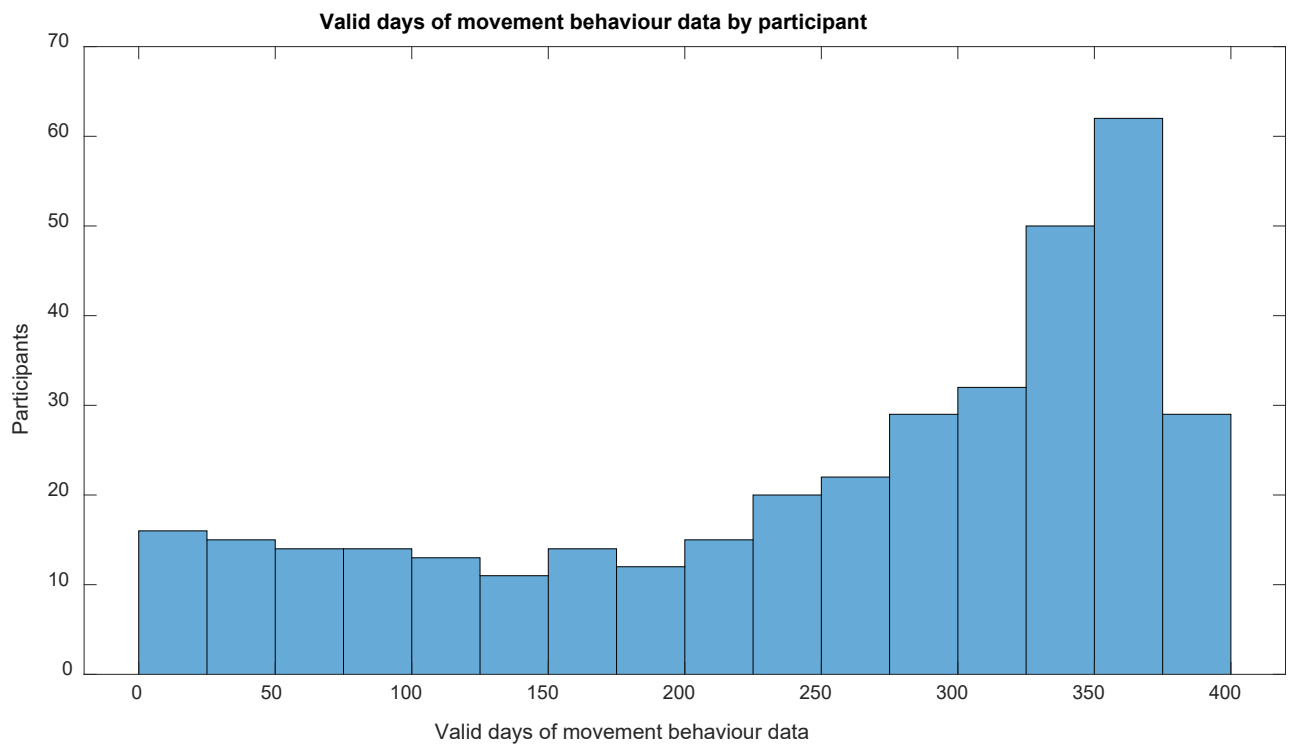

Supplement: Supplementary file 1 — Additional file 1. [file 12966_2023_1414_MOESM1_ESM.pdf]
