# Supplementary material for: Weather associations with physical activity, sedentary behaviour and sleep patterns of Australian adults: a longitudinal study with implications for climate change
Source: Int J Behav Nutr Phys Act. 2023 Mar 14;20:30. doi: 10.1186/s12966-023-01414-4 (PMC10012316; doi:10.1186/s12966-023-01414-4)
Supplement: Supplementary file 2 — Additional file 2: Supplementary Table 1. Descriptive daily weather statistics. [file 12966_2023_1414_MOESM2_ESM.pdf]

Supplementary Table 1: Descriptive daily weather statistics

	Mean	SD	Min	Median	Max	IQR	Range
Min temperature (ºC)							
Overall	11.3	4.5	0.4	11.0	31.6	5.4	31.2
Wave 1	11.4	4.9	0.04	20.5	31.6	5.9	31.6
Wave 2	11.2	4.1	1.78	11.0	26.4	4.9	24.6
Max temperature (ºC)							
Overall	21.3	6.6	8.4	20.4	43.5	9.5	35.1
Wave 1	21.6	6.9	9.5	11.2	43.5	9.9	34.0
Wave 2	21.2	6.1	8.4	20.6	41.7	9.6	33.3
Rainfall (mm)							
Overall	1.5	3.8	0	0.04	33.2	0.8	33.2
Wave 1	1.4	3.9	0	0.04	32.2	0.7	32.2
Wave 2	1.4	3.7	0	0.04	33.2	1.0	33.2
Wind speed (km/h)							
Overall	23.1	6.8	9.3	22.3	46.0	8.9	36.7
Wave 1	23.2	7.0	9.3	22.7	43.2	9.8	33.9
Wave 2	23.2	6.8	10.8	22.1	45.7	8.2	34.9
Sunshine (h)							
Overall	7.8	3.9	0	8.5	13.9	6.1	13.9
Wave 1	7.7	3.9	0	8.35	13.8	6.3	13.8
Wave 2	8.1	3.8	0	8.6	13.9	5.8	13.9
Cloud cover (/8)							
Overall	4.4	2.4	0	4.8	8.0	4.3	8.0
Wave 1	4.5	2.5	0	4.8	8.0	4.3	8.0
Wave 2	4.3	2.3	0	4.5	8.0	4.0	8.0

Notes: Overall = 1/12/2019 to 31/12/2021, Wave 1 = 1/12/2019 to 31/12/2020, Wave 2 = 1/12/2020 to 31/12/2021. IQR = interquartile range, max = maximum, min = minimum, SD = standard deviation.
